# Wrist arthroscopy for diagnosis and treatment of acute and chronic conditions

**DOI:** 10.1051/sicotj/2022015

**Published:** 2022-05-19

**Authors:** Franck Atlan, Tamir Pritsch, Daniel Tordjman, Nathan Khabyeh-Hasbani, Dania Halperin, Shai Factor

**Affiliations:** Hand Surgery Unit, Department of Orthopaedic Surgery, Tel Aviv Medical Center, 6 Weitzman Street, Tel Aviv 6423906, Israel, affiliated with the Sackler Faculty of Medicine, Tel Aviv University Tel Aviv Israel

**Keywords:** Arthroscopy, Wrist, Surgery, Diagnosis, Fracture

## Abstract

Wrist arthroscopy is a constantly evolving procedure. Allowing direct visualization and dynamic testing of intra-articular structures led to a novel approach toward traumatic and degenerative lesions based on most of the classifications routinely used in wrist surgery. The development of specific instrumentation, combined with a novel understanding of the local anatomy, progressively allowed wrist surgeons to describe more ambitious and complex surgeries. Wrist arthroscopy has become an increasingly useful tool in hand and wrist surgeons’ panoply and seems promised to have further development in the future. This paper discusses the surgical technique and the various pathologies that can be treated by arthroscopy of the wrist.

## Introduction

More than 40 years since wrist arthroscopy was first described by Chen, that reported the results of the arthroscopic assessment of the wrist in 90 patients [1]. The use of arthroscopy gained popularity during the 1980s after organized workshops developed throughout the United States. Since then, both indications and techniques have tremendously evolved [2]. Following the encouraging path seen on larger joints, the ability to enjoy a direct visualization of intraarticular structures, and to assess them dynamically, was particularly attractive in the wrist, a complex and tight joint in which eight bones articulate within each other into a tight and complex network of intra and extra-articular ligaments. Initially mostly used as a diagnostic tool [3], the development of specific instrumentation, combined with a novel understanding of the local anatomy, progressively allowed wrist surgeons to describe more ambitious and complex surgeries. Thus, wrist arthroscopy must not be considered the procedure by itself but rather as a technical tool allowing to perform various procedures in a mini-invasive manner and with improved precision.

**Table 1:** Wrist arthroscopy main indications summary.

Diagnostic arthroscopy
Acute conditions
Distal radius Fracture
Scaphoid fracture
Scapho-Lunate injuries
Traumatic TFCC lesions
Septic arthritis
Chronic conditions
Ganglion Cysts
Degenerative TFCC lesions
Scaphoid non-union
Ulnar Impaction
Degenerative and post-traumatic arthritic changes
Arthrolysis

TFCC, Triangular Fibrocartilaginous Complex.

## Setup

**Figure 1:**
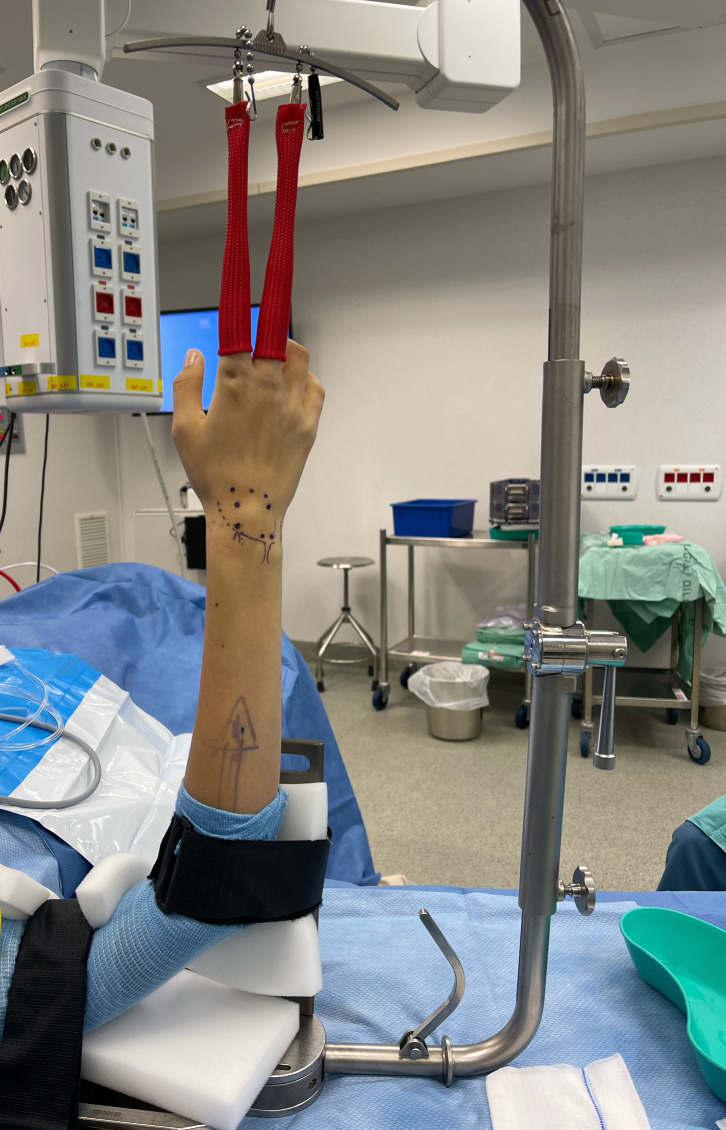
A specific wrist arthroscopy tower is usually used, providing vertical distraction from the hand table upwards.

Wrist arthroscopy follows general rules unrelated to its indication. The patient is usually supine. Traction is critical to ensure proper visualization and facilitate an atraumatic penetration of the joint. A specific wrist arthroscopy tower is usually used, providing a vertical distraction from the hand table upwards (Figure 1). The instrumentation is specifically designed for small joints, and an arthroscope of 2.7 mm or even 1.9 mm, with a 30-degree visualization angle, is generally used. Surgery can be performed under general, locoregional, or even local anesthesia, with a tourniquet on the arm. The recent advancement of Wide-Awake Local Anesthesia No Tourniquet (WALANT), as described by Lalonde [4] has revolutionized the field of hand surgery, and its use has been described in wrist arthroscopy [5]. However, the discomfort caused by the arthroscopic tower and muscular tonus compromising joint distraction make general and locoregional anesthesia relevant, especially for long and complex procedures. Wrist arthroscopy can be performed dry or with saline irrigation. Dry arthroscopy has become increasingly popular as it allows a proper visualization despite blood oozing and avoids extra-articular fluid extravasation and intra-articular synovial swelling [6]. However, a saline inflow is usually connected to the arthroscope sheath for occasional washing of blood and debris.

### Portals

**Figure 2:**
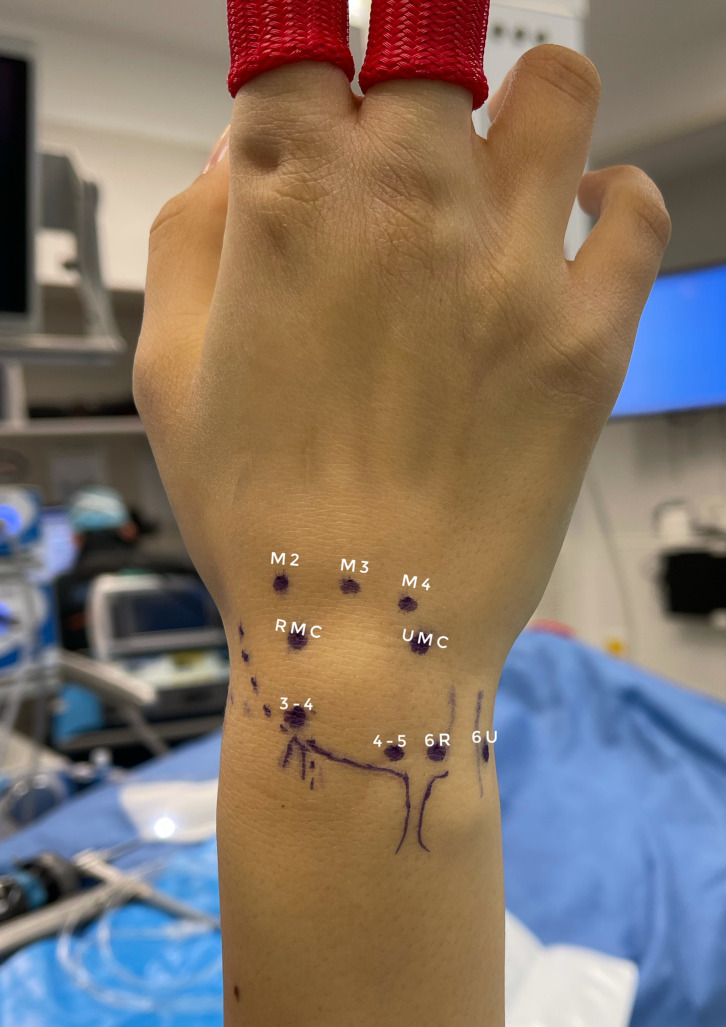
Classic portals in wrist arthroscopy. 3–4 portal is usually the first portal to be opened. It lays on the soft spot, 1 cm distal to the Lister’s tubercle, between the third and fourth extensor compartments. 4–5 portal is located between the fourth and fifth extensor compartments. 6R and 6 U portals are radial and ulnar to the sixth extensor compartment, respectively. The RMC portal lays distal to the 3–4 portal in the axis of the basis of the second metacarpal, whereas the UMC portal lays distal to the 4–5 portal in the direction of the basis of the fourth metacarpal. RMC, Radial Mid-Carpal; UMC, Ulnar Mid-Carpal (m); Metacarpal.

Due to proximity to tendons, wrist extrinsic ligaments, and neurovascular structures, and considering the small size of the surgical field, precise positioning of the portals is of paramount importance to avoid iatrogenic injuries and ensure a proper vision and instrumental range of motion. Several classic portals have been described to penetrate both radiocarpal and midcarpal joints [6]. Most of them are dorsal, as the joint is more superficial, and there is no major neurovascular structure on that level (Figure 2). At least two portals are always required for each joint, one used for the arthroscope and the other for a working tool (probe, shaver, vaper, grasper, or punch). Both can be exchanged as much as needed to allow better access and/or better visualization of the surgical field. Once the hand is installed in the arthroscopic traction tower, anatomical landmarks are drawn on the skin: the basis of the second, third, and fourth metacarpals, distal radius dorsal margin, Lister’s tubercle, ulnar and radial border of the Extensor Carpi Ulnaris, and midcarpal joint (both at the basis of the second and fourth metacarpals).

Radiocarpal portals are named according to their relationship to the extensor tendons compartment. Radiocarpal arthroscopy is used to access the distal radius’s articular surface, the Triangular Fibrocartilaginous Complex (TFCC) ligament, and the proximal articular surface of the scaphoid, lunate, and triquetrum, and the scapholunate and lunotriquetral ligaments. It can also be used to address intra-articular benign tumors and foreign body excision and for contracture release. 3–4 portal is typically the initial portal to be opened. It lays on the soft spot distal to the Lister’s tubercle, between the third and fourth compartment of the extensors (extensor pollicis longus and extensor digitorum communis). A superficial puncture wound is made on the skin with an 11 or a 15 blade, and blunt dissection is progressively made toward the joint using Stevens’s scissor or Mosquito pliers, until penetration into the joint. The natural ulnar inclination and volar tilt of the articular surface of the distal radius must be recalled to avoid iatrogenic cartilage injuries. Penetration must be smooth, and no strong resistance should be met, otherwise indicating a false route. Once the capsular joint has been clearly opened, the arthroscope sheath is inserted through the portal with a blunt trocar, then replaced by the camera itself. 4–5 portal is located in the middle of the extensor digitorum communis and the extensor digiti minimi compartment. It is usually made under arthroscopic control through the 3–4 portal, using a 22-gauge needle to previsualize the point of penetration into the joint. 6R portal (radial to the ECU compartment) and occasionally 6U (ulnar to the ECU compartment, closer to the ulnar neurovascular bundle) can also be used, further away from the 3–4 portal allowing more latitude in the motion of both the camera and the surgical tool.

Midcarpal arthroscopy is used to evaluate the cartilage of the distal and proximal carpal rows (hamate, capitate, and even trapezium and trapezoid at the STT joint) and dynamically assess the tension of the Scapholunate and luno-triquetral interosseous ligaments (SLIL and LTIL). It can also be used to address intra-articular benign tumors and foreign body excision. Two portals are usually used to access the midcarpal joint: The Radial Mid-Carpal (RMC) and the Ulnar Mid-Carpal (UMC). The RMC portal lays distal to the 3–4 portal in the axis of the basis of the second metacarpal, whereas the UMC portal lays distal to the 4–5 portal in the direction of the basis of the fourth metacarpal.

Other portals have been described for specific indications [7]. Volar portals: The Volar Radial (VR) portal allows volar access to the radial aspect of the radiocarpal joint. This portal presents specific iatrogenic risks as the radial artery and its branch passes near it. The Volar Ulnar (VU) portal allows volar access to the ulnar aspect of the radiocarpal joint. Specific iatrogenic risks concern the ulnar nerve and arterial branches. First Carpo Metacarpal (CMC1) Portals: 1R and 1U portals, respectively radial and ulnar to the first extensor compartment, are used to access the CMC joint. Those portals present a specific iatrogenic risk to the branches of the sensitive radial artery and nerve (especially 1R). A third, the Thenar portal, is also used for volar access to the CMC1 joint.

### Wrist arthroscopy indication

#### Acute conditions

Wrist arthroscopy serves as a valuable tool in acute traumatic conditions as it may allow a precise intra-articular assessment of both bony and ligamentous lesions, including dynamic testing of interosseous radiocarpal and intra carpal ligaments.

#### Distal radius fracture

The use of arthroscopy in the diagnosis, management, and treatment of distal radius fractures (DRF) has become an instrumental tool. Though arthroscopy offers an unparallel view of intra-articular pathology, the use of arthroscopy in the overall treatment of DRF remains controversial and is in continual evolution. In patients over 65 years old, DRF is the most common fracture of the upper extremities [8]. For the correction of DRFs, plating is the most common surgical treatment method [9]. For young adults and high-demand middle-aged patients, in which intra-articular involvement due to high energy trauma (with the possibility of associated soft tissue injuries is suspected), wrist arthroscopy for DRFs has been continuously preferred [10].

Abe and Fujii developed a surgical technique, plate pre-setting arthroscopic reduction technique (PART), to facilitate wrist arthroscopy during plate fixation [11]. Similar to other benefits of wrist arthroscopy, PART also permits the detection of displaced intra-articular fracture fragments, concomitant soft tissue injuries, and iatrogenic screw protrusion. One of the main indications for arthroscopy in DRFs is an intra-articular step or gap from 1 to 2 mm following closed reduction [12]. Other indications include comminuted intra-articular fractures and\or die-punch fragments, suspecting TFCC lesion, associated carpal bone fractures, intrinsic ligament injuries, widening of the distal radioulnar joint (DRUJ), and radial styloid fractures (incomplete greater arch lesion) [13].

Over the years, many studies and reviews have examined the advantages of arthroscopy in the diagnosis and treatment of DRFs. For example, one study showed that wrist arthroscopy in the use of the diagnosis of DRF was able to better detect minor gaps or step off in the articular surface, not seen by Fluoroscopy or plain radiograph [14]. A retrospective study [15] compared arthroscopy assisted versus fluoroscopically assisted reduction and external fixation of DRF. The study illustrated those patients who underwent external fixation and underwent arthroscopic assisted pinning significantly improved supination compared to those who underwent external fixation and then fluoroscopically assisted reduction and pinning. Arthroscopic assisted reduction also improved wrist extension and flexion compared to those who underwent fluoroscopic assisted reduction. Another study illustrated that after arthroscopic surgery to correct intra-articular fracture of the distal radius, 17 out of the 18 patients were able to return to work within 3 to 6 months following surgery [10].

#### Scapho-lunate injuries

The introduction of arthroscopy has enabled the acute and chronic correction of scapholunate interosseous ligament (SLIL) tear. Depending on the age of the lesion, the tear can be classified as either acute (less than six weeks), sub-acute (six weeks to three months), or chronic (more than three months). Scapholunate ligament tear is most often due to trauma with the wrist in supination and extension [16]. Such a tear may lead to chronic instability and osteoarthritis, with or without concomitant fracture of the distal epiphysis of the radius or the scaphoid. Although open reconstruction or repair for the correction of scapholunate ligament tear is feasible, with the possibility of wrist stiffness, the advancement in arthroscopy has been a pivotal technique used in the diagnosis, management, and treatment of this injury [17].

**Figure 3:**
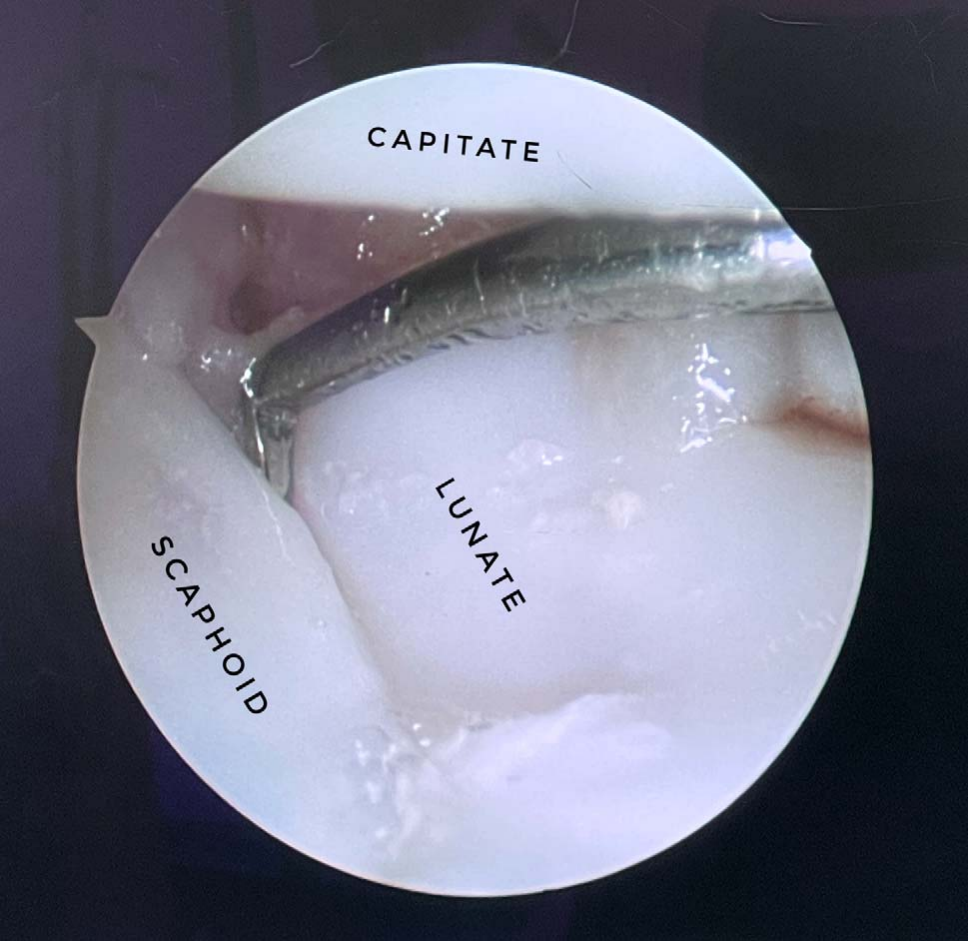
The Geissler classification [19] is commonly used to assess carpal instability and SL injuries. This figure shows a 3–4 portal visualization of the SL, with a presence of a gap between carpal bones that is less than the width of the probe, representing grade 2 of interosseous ligament injury according to Geissler’s classification. SL, Scapho-lunate ligament.

The benefits of wrist arthroscopy compared to open procedures permit the direct visualization of the cartilage surfaces, interosseous ligament, synovial tissue, and the avoidance of the exposure of tendons, joint capsule, and extensive devascularization, especially for the management of both acute and chronic injuries to the SLIL. The Dorsal Capsular Scapholunate Septum, which was described by Van Overstraeten et al. [18], clarified the anatomic basis to make arthroscopic repair conceptually valid and its importance in scapholunate instability. Most importantly, wrist arthroscopy allows for the dynamic testing of SLIL injury and, if needed, a pre-operative adjustable surgical plan. Wrist arthroscopy is especially useful in distinguishing different injuries to the SLIL. In 1996, Geissler et al. [19] developed an arthroscopic-based grading and classification system for carpal interosseous tears. (Figure 3) Expansion of Geissler’s grading system includes the European Wrist Arthroscopy Society’s more thorough categorization that includes the site of the SLIL injury [20]. With the innovation of wrist arthroscopy, the number of passes to correctly position the KW is minimized, leading to the minimally invasive yet accurate correction of SLIL tear. Compared to acute injury of the SLIL, management of chronic injury to the SLIL is controversial. Treatment options using arthroscopy include debridement, ligamentous reconstruction, proximal row carpectomy, intercarpal fusion, and salvage procedures such as wrist fusion.

#### Traumatic lesions of the triangular fibrocartilaginous complex (TFCC)

**Figure 4:**
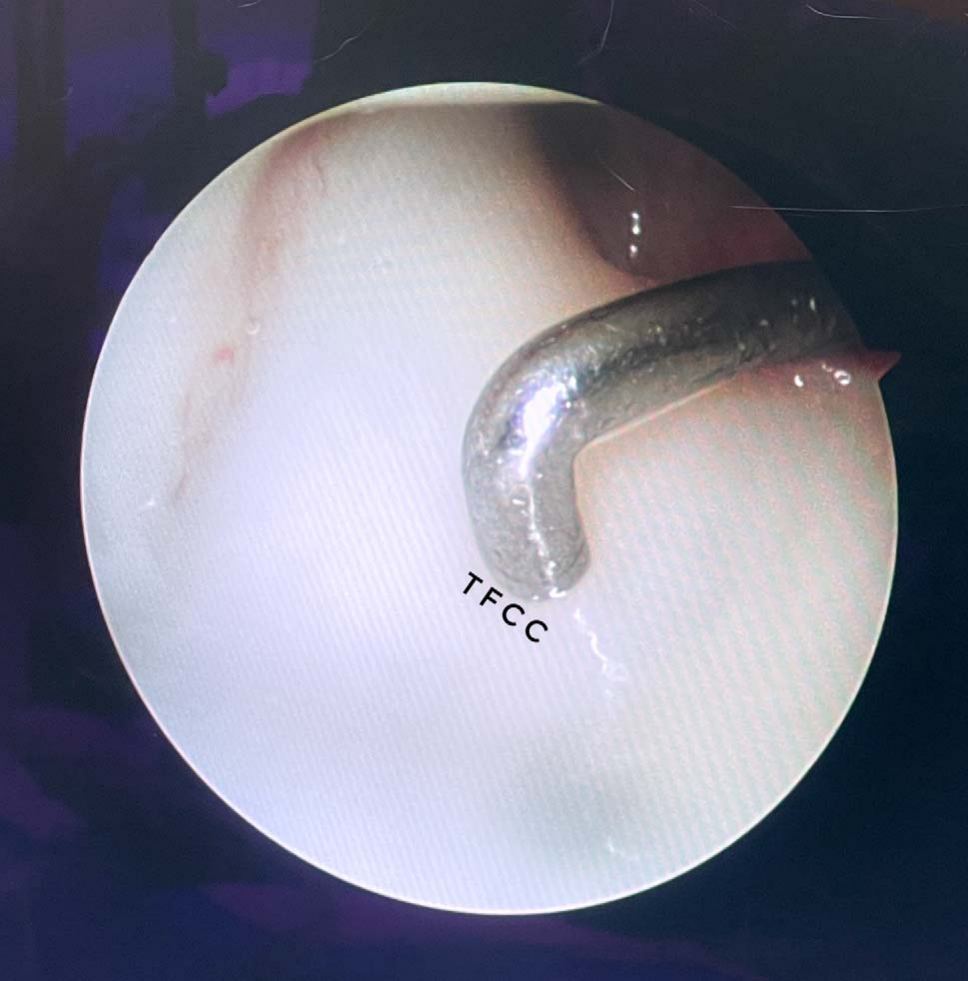
Arthroscopy visualization of the TFCC allows direct and magnified visualization of the anatomical area, which provides the surgeon a more thorough understanding of the injury compared to an open procedure. TFCC, Triangular Fibrocartilaginous Complex.

The TFCC serves as a stabilizer of the distal radioulnar joint, which provides unrestricted pronation and supination, which is vital to performing more complicated and sophisticated tasks [21]. During the past three decades, increased knowledge of the anatomy and pathophysiology of the TFCC has led to better diagnosis, treatment, and long-term recovery from such injury [22]. Because of its pivotal role in functionality, rotation and load-bearing, and important anatomical location, the TFCC is highly prone to injuries and attritional wear [23]. Traumatic tears may occur following a fall with the wrist in hyperextension or rotational injuries [24]. Therefore, lesions of the TFCC may be due to trauma or degeneration with increasing age. To diagnose such injury or lesion, patients with a history of ulnar-sided wrist pain that increases during the powerful rotatory hand (i.e., pushing down on a doorknob) [21]. Compared to other forms of imaging modalities, wrist arthroscopy is considered the “gold standard” in TFCC injuries diagnosis [25]. Arthroscopy of the wrist for suspected TFCC injury allows for direct, magnified visualization of the anatomical area, which gives the surgeon a more thorough understanding of the injury than an open procedure (Figure 4).

With the use of wrist arthroscopy, the Palmer classification [26] categorizes TFCC injuries as either traumatic or degenerative based on the location of the injury and the nature of their formation.

The TFCC is inspected through the 3–4 portal, in which a probe is inserted through either the 4–5 or 6-R portal to scan the TFCC surface for tears. Specific tests, such as the trampoline and hook test, help evaluate peripheral tear and the integrity of the deep fibers of the TFCC, respectively [27]. For degenerative TFCC tears, debridement of frayed edges using wrist arthroscopy may suffice. Arthroscopic foveal repair of the TFCC has been proven to restore DRUJ stability with satisfactory results [28].

In a retrospective study performed by Hahn et al., TFCC lesions were identified during arthroscopy in 83% of the patients, while MRI showed an average sensitivity of 69% and a specificity of 60% [29].

#### Septic Arthritis of the wrist

Bacterial septic arthritis is a joint-threatening emergency associated with considerable morbidity and mortality [30]. When septic arthritic occurs in the upper extremity, it is estimated that 23% of the cases involve the wrist [31].

To properly diagnose septic arthritis, the patient’s history and physical examination performed by the physician is an instrument and is usually supported by laboratory studies and imaging. Typically, patients with septic arthritis of the wrist present with systemic complaints including fever, chills, sweats, and more specifically, an erythematous, swollen and painful joint with limited motion.

According to a 2009 study that compared arthroscopic versus open irrigation and debridement of septic arthritis of the wrist [32], arthroscopic treatment was found to be more effective, with patients having fewer operations and a shorter hospital stay. Under general anesthesia and after inspection and debridement, a complete wrist joint irrigation using three liters of normal solution can be performed through the radiocarpal and midcarpal joints.

### Chronic conditions

#### Ganglion cysts of the wrist (volar and dorsal)

**Figure 5:**
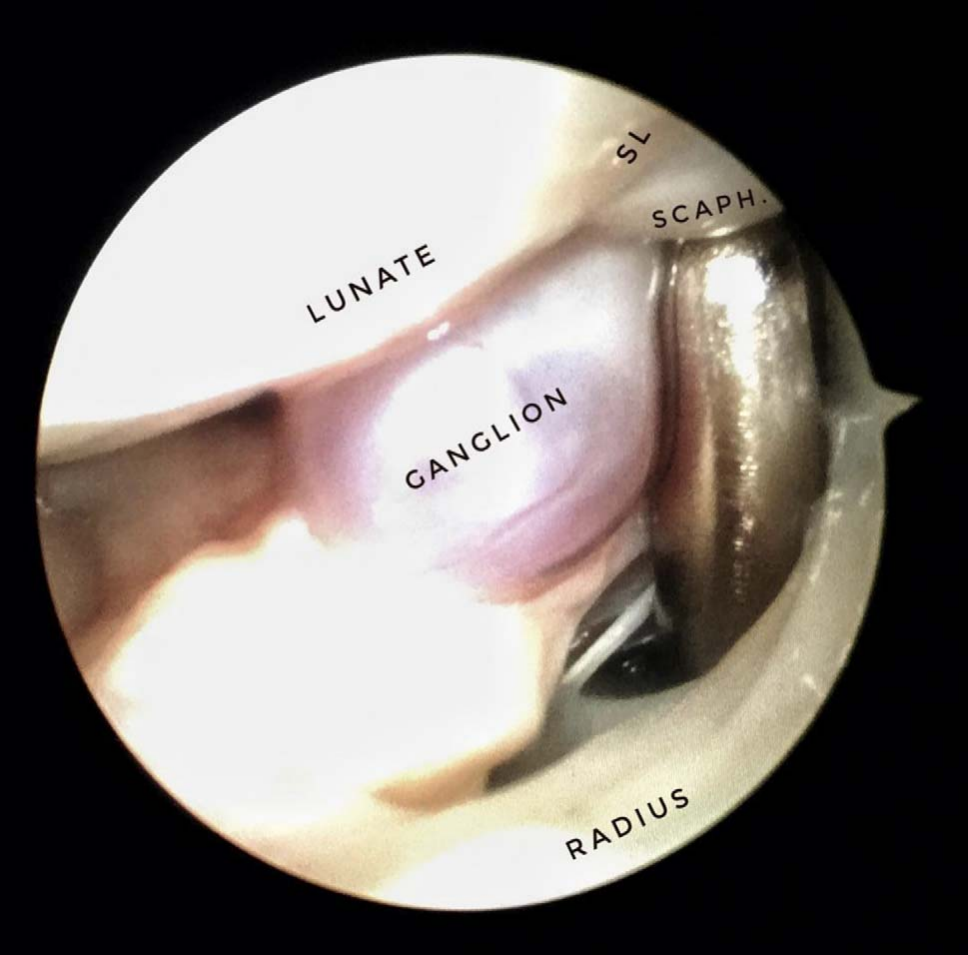
Arthroscopic view ganglion originates from the SL. Arthroscopic excision of ganglion cyst allows the surgeon to visualize the origin of ganglion and to remove it from inside while also avoiding scaring and ensuring that the origin is removed. Benefits of the procedure include direct visualization of the articular surfaces and stability of the intrinsic ligaments, less surgical dissection, less postoperative pain, faster recovery, and earlier return to work and activities of daily living. SL, Scapho-lunate ligament; SCAPH, Scaphoid.

Ganglion cysts are usually treated by an open incision which leads to scar tissue as well as limitation of motion at an early stage [28]. Arthroscopic resection of dorsal carpal ganglion cysts has become increasingly popular in recent years. Using arthroscopy, the origin of the ganglion can be visualized and removed from the inside, thus avoiding scaring and ensuring that the origin of the ganglion is removed [22] (Figure 5). Arthroscopic resection, using the 3–4, 4–5, and 6U portals, is made with little morbidity [33]. The benefit of the procedure is that scars are unnoticeable and wrist mobility and strength reach a close to normal range within three months. Other advantages include less postoperative pain and an earlier return to work [34].

#### Ulnar sided pain wrist

##### Diagnostic arthroscopy

Ulnar-sided pain wrist is a frequent cause for consultation in hand surgery. The main causes are TFCC lesions, ulno-triquetral tears, Extensor Carpi Ulnaris (ECU) tendinitis or instability, piso-triquetral cyst, or non-specific overuse of the hand. Wrist arthroscopy can be a useful diagnostic tool in case of persisting ulnar-sided wrist pain with inconclusive MRI and may allow simultaneous treatment of the cause if found.

##### Degenerative TFCC lesions and ulno-triquetral tear

At the difference of acute traumatic TFCC tear, which may be treated by repair through reinsertion in some cases, degenerative TFCC lesions require debridement and, whenever possible, treatment of the cause, for example, in the case of distal radius fracture malunion with positive ulnar variance. Instruments are used to debride the damaged edges back to the stable rim, remove injured tissue, and assess associated mirror chondral damage on the lunate or lunotriquetral ligament tear. Clinically significant improvements were reported in pain, function, and grip strength [35, 36].

##### Scaphoid non-union

Scaphoid non-union leads to wrist arthritis through a predictable pattern called Scaphoid Non-Union Advanced Collapse, or SNAC wrist [37]. Treatment of scaphoid non-union relies on the basic principle that applies for every non-union in orthopedics: debridement of the non-union site, reduction of the fracture, bone grafting, and stabilization [38]. Conventional open approaches lead to reliably good chances of achieving bone healing but require an extensive approach, potentially damaging the scarce blood supply of the scaphoid and often implying postoperative stiffness [39]. Arthroscopically assisted methods have been described with encouraging results to avoid jeopardizing periosteal vascularization of the scaphoid and deep scaring of the surrounding soft tissues [40]. Through midcarpal portals, the non-union site can be visualized and debrided, a bone graft can be inserted, and proper reduction and stabilization of the scaphoid can be ensured. Potential osteochondral damages can be assessed, and a radial styloidectomy can be performed.

##### Ulnar Impaction

Ulnar impaction is a common complication after distal radius malunion. In its most common form (following Colles fracture), distal radius malunion consists of a radial, dorsal, and proximal collapse of the distal epiphysis of the radius, leading to a positive ulnar variance. This can cause an impaction between the ulnar head and the lunate, often associated with degenerative TFCC injury. This condition, known as ulnar impaction, can be treated either by distal radius corrective osteotomy if there is significant dorsal and or radial angulation associated with radial collapse or by ulnar shortening if the ulnar impaction is the main problem. Ulnar shortening can be achieved through a shaft shortening osteotomy if the ulnar variance is more than 4 mm positive or through an intra-articular resection of the distal part of the ulnar head associated with debridement of the TFCC if it is less than 4 mm. This latter option, known as the Wafer procedure, can be performed arthroscopically, with good results and fewer complications compared to ulnar shortening osteotomy.

##### Arthritic changes in the wrist

**Figure 6:**
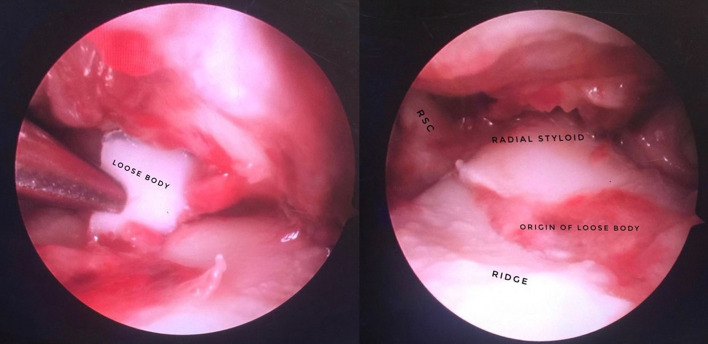
Arthroscopic view of a loose body in radio-carpal joint and its origin that lead to osteochondral defect in the articular surface of the radius.

Wrist arthroscopy has shown extreme sensitivity to detect chondral lesions and assess degenerative changes [3] (Figure 6). Beyond its diagnostic value, it also allows therapeutical procedures. Arthroscopic synovectomy has been described in degenerative arthritic wrist following Scapholunate advanced collapse (SLAC) or SNAC, as well as in the rheumatoid arthritic wrist, both showing encouraging results [41].

Bone resection and cartilage resection in preparation for partial or total wrist arthrodesis can also be performed arthroscopically, with satisfying results. Pinal et al. reported a series of arthroscopic resection arthroplasty for malunited intra-articular distal radius fractures. At 6 months follow-up, improvements in functional scores and range of motion were observed.

##### Arthroscopic arthrolysis

Stiffness is a common complication observed secondary to trauma, especially in case of prolonged immobilization. Both intra-articular and extra-articular structures can be involved [42].

Arthroscopic arthrolysis of the wrist has shown good and sustainable results restoring range of motion and diminishing pain. Hattori et al. described a fibrotic band frequently observed between the scapholunate ligament and the ridge of the articular surface of the radius between the lunate fossae and the scaphoid fossae [43]. Both dorsal and volar capsules can be carefully released if intra-articular release is not enough, but care must be taken to preserve extrinsic radiocarpal ligaments, especially the Long Radio Lunate ligament, to avoid ulnocarpal translation.

## Conclusion and future perspectives

Wrist arthroscopy is a constantly evolving field. Allowing direct visualization and dynamic testing of intra-articular structures led to a novel approach toward traumatic and degenerative lesions based on most of the classifications routinely used today in wrist surgery.

Thanks to the development of appropriate tools, a better understanding of wrist anatomy and pathology, and a growing experience worldwide, this technique, initially mostly used for diagnostic purposes, now allow therapeutical procedures with the result either as good or better compared to open procedures, both in acute traumatic and chronic degenerative conditions. The development of smaller arthroscopes (1.9 mm) and the description of safe portals are now allowing arthroscopy of smaller joints in hand, such as first carpometacarpal, metacarpophalangeal, and even interphalangeal joints. Indications vary from debridement and synovectomy to arthroscopically assisted repairs, often realized under locoregional or even WALANT local anesthesia. That technical progress simplified the logistics surrounding arthroscopy, allowing its implementation into virtually any kind of surgical practice.

Wrist arthroscopy has become an increasingly useful tool in hand and wrist surgeons’ panoply and seems promised to further development in the future.
